# Nutrient Intake through Childhood and Early Menarche Onset in Girls: Systematic Review and Meta-Analysis

**DOI:** 10.3390/nu12092544

**Published:** 2020-08-22

**Authors:** Ngan Thi Kim Nguyen, Hsien-Yu Fan, Meng-Che Tsai, Te-Hsuan Tung, Quynh Thi Vu Huynh, Shih-Yi Huang, Yang Ching Chen

**Affiliations:** 1 School of Nutrition and Health Sciences, Taipei Medical University, Taipei 110, Taiwan; kimngan1702@gmail.com (N.T.K.N.); d07849004@ntu.edu.tw (T.-H.T.); 2 Department of Family Medicine, Taipei Medical University Hospital, Taipei 110, Taiwan; derossi83621@gmail.com; 3 Department of Pediatrics, National Cheng Kung University Hospital, College of Medicine, National Cheng Kung University, Tainan 704, Taiwan; ache93@yahoo.com.tw; 4 Department of Pediatrics, Faculty of Medicine, The University of Medicine and Pharmacy at Ho Chi Minh City, Ho Chi Minh City 700000, Vietnam; quynh.huynh@ump.edu.vn; 5 Graduate Institute of Metabolism and Obesity Sciences, Taipei Medical University, Taipei 110, Taiwan; 6 Nutrition Research Center, Taipei Medical University Hospital, Taipei 110, Taiwan; 7 Department of Family Medicine, School of Medicine, College of Medicine, Taipei Medical University, Taipei 110, Taiwan

**Keywords:** puberty, menarche, sexual maturation, nutrient intake, childhood, meta-analysis

## Abstract

Among the genetic and environmental risk factors, nutrition plays a crucial role in determining the timing of puberty. Early menarche onset (EMO) is defined as when girls reach menarche onset at an age which is earlier than the mean/median age of menarche, between 12 and 13 years of age, according to individual ethnicity. The present study examined the association between nutrient intake in childhood and EMO risk in healthy girls by performing a systematic review and meta-analysis of prospective studies. We screened EMBASE, Cochrane Library, PubMed/MEDLINE, and Web of Science databases for 16 eligible studies with all medium-to-high quality scores ranging from 3 to 5 of 6 possible points with 10,884 subjects. Higher intakes of energy (risk ratio (RR) = 3.32, 95% confidence interval (CI) = 1.74–6.34, *I*^2^ = 97%), and protein (RR = 3.15, 95% CI = 2.87–3.44, *I*^2^ = 0%) were associated with EMO risk. For each additional 1 g/day animal protein intake in childhood, the age at menarche was approximately two months earlier (β = −0.13, *I*^2^ = 55%), and high iron intake was associated with EMO (RR = 1.20, 95% CI = 1.03–1.40, *I*^2^ = 0%). Polyunsaturated fatty acid (PUFAs) intake was associated with EMO risk with a dose-response effect (RR = 1.25, 95% CI = 1.05–1.49, *I*^2^ = 44%). Girls with a high intake of fiber and monosaturated fatty acids (MUFAs) in childhood experienced later menarche onset (RR = 0.83, 95% CI = 0.69–1.00, *I*^2^ = 31%; RR = 0.66, 95% CI = 0.50–0.86, *I*^2^ = 0%, respectively). Thus, adherence to a high intake of animal proteins-, iron- and PUFA-rich food diet makes girls more likely to have EMO, while a high intake of fiber- and MUFA-rich foods may protect girls from EMO. Further studies are expected to investigate the role of specific types of PUFAs and MUFAs on EMO to promote healthy sexual maturity in girls.

## 1. Introduction

Since the late 19th century, early puberty onset, defined by a decreased mean age at menarche (AAM), has been noted as a global trend [1]. Menarche, the first menstrual period, is a widely used variable in epidemiological studies because it is a clear sign, is well-recalled, and occurs relatively late, between 12 and 13 years of age, and after the onset of breast development and the growth spurt in girls [2,3]. Individuals with earlier menarche onset (EMO) are at higher risk of hormone-related cancers [4,5], cardiovascular diseases [6], and all-cause mortality in later life [7]. The awareness of modifiable factors of EMO is thus critical to public health implications.

Of the genetic and environmental risk factors, nutrition plays a crucial role in determining the timing of puberty [1]. Caloric over-consumption may lead to EMO due to its contributions to the accumulation of body fat, which elevates leptin levels, a signal to the brain prompting puberty onset [8,9]. Therefore, sexual maturation is sensitive to nutritional regulation that requires the appropriate dietary intake for healthy pubertal growth. Investigations of nutrient intake in association with EMO might provide valuable information for further research on exploring the mechanism. Moreover, nutrient-based food groups or dietary pattern recommendations could be provided for children who have undergone pubertal growth.

Nutrient-based research serves to disseminate knowledge about essential nutrients that are important when making recommendations tailored to at-risk populations, such as EMO girls [10]. The fact that the associations of a prospective intake of energy [11,12], fat [13,14,15], fiber [16,17,18], and protein, including animal and vegetable protein [17,19] and EMO, remain controversial [1]. Although total fat intake in childhood has been reported to influence EMO [13,20], other findings have shown no clear evidence of the association between fat consumption and EMO [15,21]. High prepubertal isoflavone intake has been linked to delaying puberty onset in girls [22]. Foods containing high amounts of isoflavones are often rich in dietary fiber [23]. However, no associations between fiber and vegetable intake with menarche onset have been reported [15,19,22]. These opposing findings may have been due to heterogeneity in study design, methodological dietary assessment, and pubertal marker (growth spurt, thelarche, pubic hair, and menarche) [24]. In light of recent findings, to our knowledge, this systematic review and meta-analysis were firstly performed to investigate the effect of as many possible nutrient intakes on EMO among healthy girls.

## 2. Materials and Methods

We conducted a protocol for the systematic review, which was registered in the PROSPERO International Prospective Register of Systematic Reviews (https://www.crd.york.ac.uk/prospero, identifier CRD42020156430).

### 2.1. Eligibility Criteria

#### 2.1.1. Definitions of EMO and Non-EMO (NEMO)

All the prepubertal girls recruited in each study were in a similar age range (Table S2). EMO was defined when girls reached menarche onset at an age that was earlier than mean/median AAM [25] (12–13 years of age [2,3]) according to individual ethnicity. For instance, the mean/median AAM of Canadian girls was 11.9 years [16,26], of American girls it was 12.5 years [13,21,27], of German girls it was 12.8 years [14,22,24], of English girls it was 12.8 years [19], of Tehranian girls it was 12.0 years [28], and of Australian girls it was 12.6 years [15]. Hence, our participants were divided into two groups according to pubertal outcomes: EMO and NEMO. The EMO group comprised girls who had menstruated before mean/median AAM in each study, meanwhile the NEMO group comprised girls who had not reached menarche.

#### 2.1.2. Inclusion and Exclusion Criteria

The PECOS (participants, exposure, comparison, outcomes, and study design) criteria (Table S1) was used to determine the eligibility of studies, the inclusion criteria was: (i) healthy prepubertal girls; (ii) nutrient intake in childhood assessed before menarche onset; (iii) nutrient intake assessment available for both EMO and NEMO groups; (iv) main outcomes were EMO and NEMO or AAM; and (v) prospective studies.

We excluded studies if (i) the children had any disease that affected pubertal development (e.g., congenital gonadal dysplasia or iodine deficiency disorder); (ii) the study was cross-sectional, a randomized control trial, an interventional study, a systematic review, or a meta-analysis; and (iii) puberty onset was defined by a growth spurt, thelarche, or pubic hair. 

### 2.2. Search Strategy

The present study searched four electronic databases up to April 2020: EMBASE, Cochrane Library, PubMed/MEDLINE, and Web of Sciences. We used a medical subject heading terms and the free terms “menarche” or “menarcheal” or “puberty” or “pubertal” or “sexual maturation” or “timing of puberty” in combination with “diet” or “dietary intake” or “intake” or “nutrition” or “consumption.” The results were limited by “case–control study” or “cohort analysis” or “comparative study” or “longitudinal study” or “observational study” or “prospective study” or “retrospective study.” The search was related to human participants who were preschool children (2–5 years), children (6–12 years), or adolescents (13–18 years). The search was also limited to English original articles.

One of our reviewers identified studies, and another reviewer screened the titles for eligibility. Both reviewers independently screened the abstracts and full-text articles. Disagreements were resolved by a third reviewer. All retrieved publications were entered into Endnote (v. 9.3, 2019, Clarivate).

### 2.3. Data Extraction

A standardized form was employed to extract data relating to the first author, publication year, country, population size, age at dietary assessment, dietary assessment method, nutrient intake, follow-up time, AAM, size of EMO and NEMO groups, confounders, and main findings. A standardized form was employed to extract data relating to the first author, publication year, country, population size, age at dietary assessment, dietary assessment method, nutrient intake, follow-up time, AAM, size of EMO and NEMO groups, confounders, and main findings.

### 2.4. Quality Assessment of Evidence

The Quality Assessment Tool for Systematic Reviews of Observational Studies (QATSO) was used [29]. Six questions could be answered with “yes” or “no” in four indicators: external validity (maximum of 1 point), reporting (maximum of 3 points), bias (maximum of 1 point), and confounders (maximum of 1 point). The total modified QATSO score could be up to 6 points (6 stars). Study quality can be divided into good (5–6 points), satisfactory (3–4 points), and poor (0–2 points), representing a low, medium, and high risk of bias, respectively.

### 2.5. Data Analysis and Statistical Methods

Mean and standard deviation (SD) were computed from studies that provided the median, range, and sample size [12,16,18,19] according to the method by Hozo et al. [30] We converted calories from megajoules and kilojoules to kilocalories (1 kcal = 4.184 kJ; = 4.184.10^−3^ MJ). Calories expressed as kcal/height [28] were converted into calories (kcal) by referencing the World Health Organization child growth standard.

Though raw data queried from the authors were not available, we extracted the exact mean and SD of nutrient intake to assess the weighted mean difference (WMD) with 95% confidence interval (CI) between the EMO and NEMO groups without adjusting confounders. Sensitivity analyses were done by the adjusted risk ratio (RR) with 95% CI for a dichotomous outcome (EMO and NEMO), and the adjusted beta regression coefficient (β) with standard error (SE) for a continuous outcome (AAM) were extracted from included studies that were controlled primarily by mother’s AAM, parent education, age, height, body weight, and total EI (Table S1). Dose-response analyses were used to estimate the risk of different quartiles of nutrient intake in relation to EMO.

Pooled estimated results were calculated using the random-effect models to reflect variability in all studies. To assess between-study heterogeneity, Cochran’s Q test was used to obtain *p* values and *I*^2^ value. An *I*^2^ > 50% or a *p* < 0.05 indicated heterogeneity across studies [31]. To detect publication bias, we used Egger’s regression test, and *p* < 0.1 has been recommended to indicate significant publication bias [32,33]. Meta-regression was performed to determine whether the baseline characteristics affected the heterogeneity across studies. Modifier factors were country (Canada, Chile, Colombia, England, Iran, Germany, Greece, Iran, Netherland, United States) [13,16,26,34], baseline body mass index (BMI) [12,13,19], age at dietary assessment (years old) [12,14,21], dietary measurement method (dietary record, food frequency questionnaire (FFQ) [13,14,16,18,22,27,28], physical activity (hours/week) [12,18,26], maternal AAM (years old) [12,18,19,28], and socioeconomic status (high, middle, low) [12,14,19,27,35]. When *p* < 0.05, subgroup analyses were applied.

All data were analyzed using the “meta” package in R software (v. 3.3.2, RStudio, Inc., Boston, MA, USA).

## 3. Results

### 3.1. Study Selection

**Figure 1:**
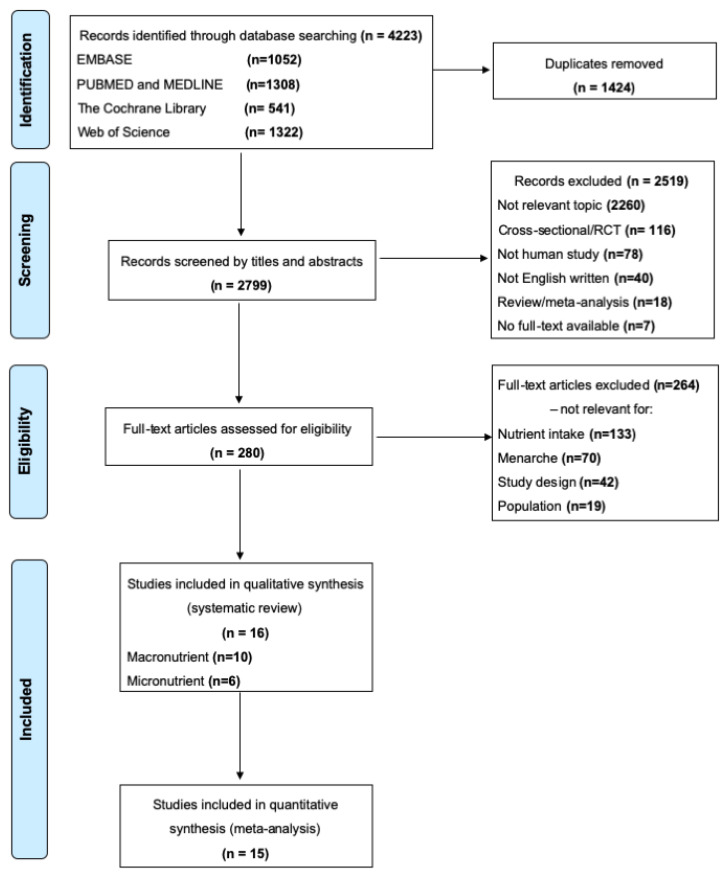
Study flow chart. *Definition of abbreviations*: EMBASE, Elservier database; RCT, randomized controlled trial.

4223 records were identified. After removing 1424 duplicates, we excluded 2519 records based on screening titles and abstracts. Of the remaining papers, 264 of 280 were removed after the full text had been assessed for eligibility. Finally, 16 longitudinal studies involving 10,884 girls were selected for systematic review and meta-analysis in the present study (Figure 1).

### 3.2. Study Characteristics

Dietary assessment was performed in early childhood (age < 8 years) in 4 studies [14,22,24] and in children aged > 8 years in 11 studies [12,16,17,18,19,20,21,26,27,28,34] (Table 1). Dietary records were used in nine studies, the forms of which were 24-h recall [28,34], 3- and 7-day records [16,17,26], and 3-day weighed records [14,24]. A food frequency questionnaire (FFQ) consisting of a semi-FFQ [12,18,19,20,21,27] or dietary history interview [13] was implemented in seven studies (Table 1).

Ten studies reported the total energy intake (EI) and macronutrient intake (carbohydrate, total protein, and total fat) in relation to EMO. A few of the studies considered the effects of monounsaturated fatty acids (MUFAs) [12,16,18,26,27], poly-unsaturated fatty acids (PUFAs) [12,16,18,19,26,27], micronutrients [16,19,26,27,28,34], animal protein [13,14,15,17,19,24], fiber [16,17,18,19,22,26,27], and isoflavones [22] on menarche onset. The mean difference in nutrient intake in childhood between the EMO and NEMO groups was analyzed through unadjusted testing [12,18,19,27,28] and adjusted testing for age, mother’s AAM, and EI [16,26,27]. The adjusted models were used to determine the association between EMO and either quartiles of nutrient intake [16,18,20,21,26,27] or tertiles of nutrient intake [14,22]. Linear regression [13,14,17] and logistic regression [19,26,28] were used (Table 1).

### 3.3. Quality Assessment

Among the 16 selected studies, 8 studies [12,16,17,18,19,22,26,28] were assessed to have low risk of bias (5–6 points) and 8 studies [13,14,15,20,21,24,27,34] to have moderate risk of bias (3–4 points) (Table S2). No study was graded as having a high risk of bias (0–2 points). In addition, no significant publication bias was found (Table S3).

### 3.4. Meta-Analysis, Publication Bias, and Sensitivity Analysis

#### 3.4.1. Association between Total EI and EMO

**Figure 2:**
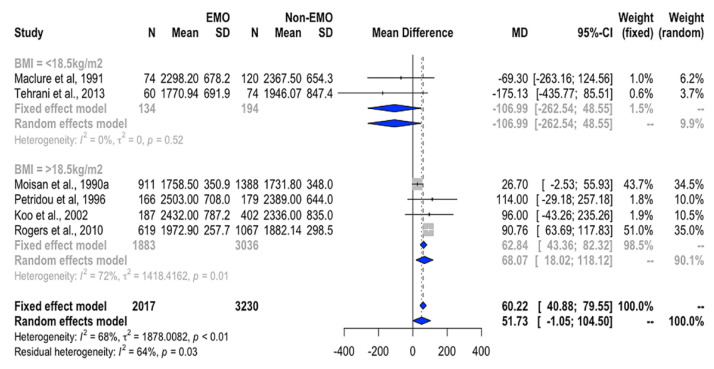
Forest plot of WMD of EI between EMO and NEMO groups stratified by BMI < 18.5 kg/m^2^ and BMI ≥ 18.5 kg/m^2^. *Definition of abbreviations:* EMO, earlier menarche onset; NEMO, non-early menarche onset; MD, mean difference; WMD, weighted mean difference.

**Figure 3:**
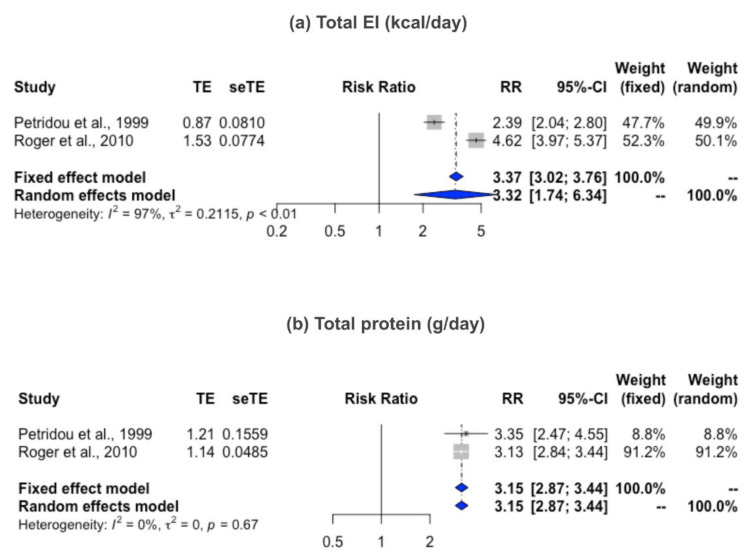
Risk ratio of menarche according to total EI (**a**) and protein intake (**b**) among EMO and NEMO girls. *Definition of abbreviation*: EI, energy intake; EMO, early menarche onset; NEMO, non-early menarche onset; RR: risk ratio.

Girls with EMO had a higher WMD of total EI than girls with NEMO with the existence of heterogeneity (*I*^2^ = 68%, *p* < 0.01, Table 2). Given that the meta-regression identified a possible effect of baseline BMI on the EI–EMO relationship (Table S4), we then performed subgroup analysis. For girls with BMI ≥ 18.5 kg/m^2^, the WMD of EI remained significantly higher in the girls with EMO than in the girls with NEMO (WMD = 68.1 kcal/day, 95%CI = 18.0 to 118.1 kcal/day, Figure 2). Additional analysis showed that the higher EI was positively associated with EMO risk (RR = 3.3, 95% CI = 1.7 to 6.3; Figure 3a).

#### 3.4.2. Association between Protein Intake and EMO

**Figure 4:**
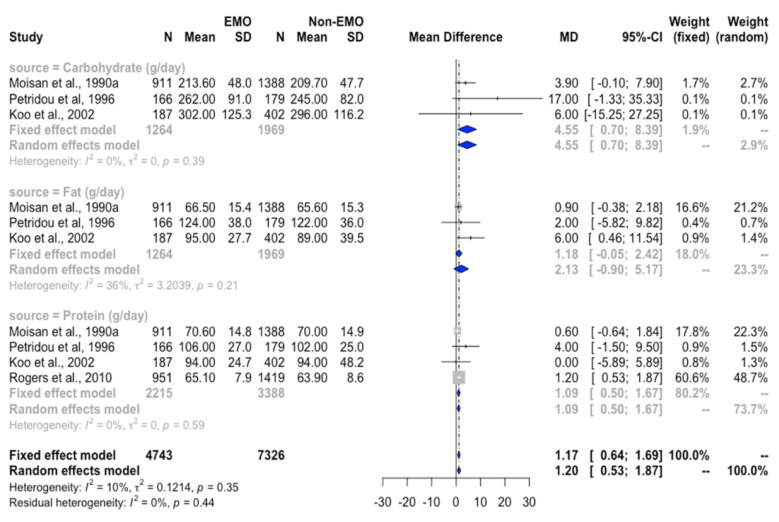
Forest plot of WMD of carbohydrate, protein, and fat intakes between EMO and NEMO groups in healthy girls with BMI ≥ 18.5 kg/m^2^. *Definition of abbreviations*: EMO, earlier menarche onset; NEMO, non–early menarche onset; MD, mean difference; WMD, weighted mean difference.

The mean difference of protein intake between girls with EMO and NEMO, as expressed by g/day or %energy, were similar. Given that BMI may be a critical factor in the meta-regression (*p* < 0.01; Table S4), subgroup analysis was performed and revealed that among girls with BMI ≥ 18.5 kg/m^2^, the higher WMD of protein was observed in the EMO group (WMD = 1.1 g/day, 95%CI = 0.5 to 1.7, *I*^2^ = 0, Figure 4). In addition, high total protein intake was linked to EMO risk (RR = 3.2, 95% CI = 2.9 to 3.4, *I*^2^ = 0%; Figure 3b). Sensitivity analysis also supported the risk of protein intake [16,21,26] to EMO (quartile 3 vs. lowest level, RR = 1.1, 95% CI = 1.0 to 1.3, *I*^2^ = 0%; Table S5).

**Figure 5:**
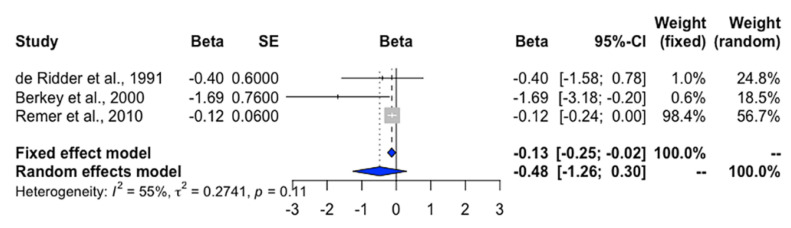
Forest plot of the beta regression coefficient of age at menarche onset for every 1 g/day increase of animal protein intake. *Definition of abbreviations*: Beta, beta coefficient; SE, standard error.

Girls with EMO had higher intake of animal protein [19] than girls with NEMO (MD = 1.4 g/day, 95% CI = 0.7 to 2.1, *p* < 0.001). Additionally, every 1-g/day increase in animal protein intake in early childhood was linked to approximately 2-month earlier AAM (Figure 5) [13,17,24].

#### 3.4.3. Association between Fat Intake and EMO 

**Figure 6:**
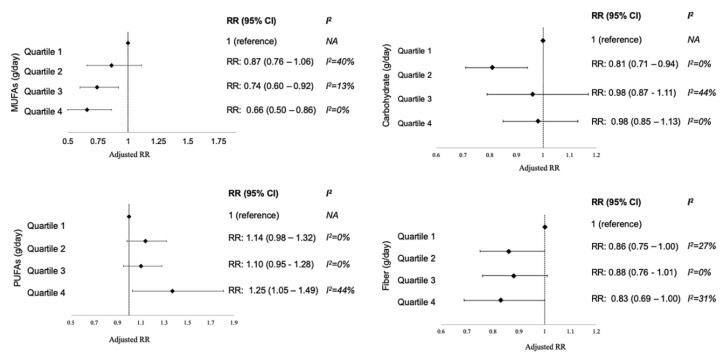
RR of menarche according to quartiles of MUFAs, PUFAs, carbohydrate, and fiber intakes among EMO and NEMO groups. *Definition of abbreviations*: CI, confidence interval; EMO, earlier menarche onset; MUFAs, monounsaturated fatty acids; NEMO, non-early menarche onset; PUFAs, polyunsaturated fatty acids; RR, risk ratio.

WMD of total fat and MUFAs between EMO and NEMO girls did not show any difference using a random effect model (Table 2). A similar result in total fat consumption could be observed by using either g/day or %energy. Girls in the EMO group consumed higher PUFAs than NEMO (WMD = 0.5 g/day, 95%CI = 0.2 to 0.8, *I*^2^ = 21%, Table 2). The associations between MUFAs and PUFAs with the EMO risk also revealed the dose-response effects (Figure 6). The higher intake of PUFAs was positively associated with EMO risk when compared with the reference level (RR = 1.25, 95%CI = 1.05 to 1.49, *I*^2^ = 44%, Figure 6). In contrast, the higher intake of MUFAs was negatively linked to EMO risk (Figure 6, Table S5) [16,18,26,27].

#### 3.4.4. Association between Carbohydrate Intake and EMO

Similar results in carbohydrate intake could be observed by using either g/day or % energy. However, high heterogeneity was found in the comparison of carbohydrate intake (g/day) between the EMO and NEMO groups (Table 2) across the included studies [12,16,18,27,28]. In the subgroup analysis, we found that in girls with BMI ≥ 18.5 kg/m^2^, the overall WMD of carbohydrate intake was higher in the EMO group than in the NEMO group (WMD = 4.6 g/day, 95%CI = 0.7 to 8.4, *I*^2^ = 0, Figure 4). Surprisingly, the higher intake of carbohydrate was linked to later menarche (quartile 2 vs. lowest quartile: RR = 0.81, 95% CI = 0.71–0.94, *I*^2^ *=* 0%; Figure 6) when considering the adjusted RR from three included studies [16,21,26].

No significant difference was found between fiber intake [17,18,27] and EMO (Table 2). However, Figure 6 reveals that the dose-response effect of fiber intake was likely linked to delay menarche onset (Figure 6).

#### 3.4.5. Association between Micronutrient Intake and EMO

Girls with EMO were more likely to consume high amounts of magnesium, vitamin C [16,27], and carotene (Table 2). The highest level of iron intake was significantly linked to EMO when compared with the lowest level (RR = 1.20, 95% CI = 1.03–1.40, *I*^2^ = 0%), and high intake of vitamin B_1_ was positively associated with EMO (RR = 1.17, 95% CI = 1.00–1.37, *I*^2^ = 0%; Table S5).

## 4. Discussion

In this systematic review and meta-analysis, 16 prospective studies involving 10,884 healthy girls were systematically investigated to determine the relationships between nutrient intake in childhood and EMO. High intakes of energy, protein, animal protein, and iron in childhood were associated with EMO. We observed the dose-response effect between PUFA intake and EMO risk and between MUFA intake and delayed menarche onset. In addition, the high intakes of fiber in childhood were likely linked to delayed menarche onset.

The present study confirmed the positive association between EI and EMO in girls with BMI > 18.5 kg/m^2^ (Figure S1 and S2), which was not identified in a previous study [1]. The fact that excess calories result in more body fat, which is positively associated with leptin levels mediating pubertal onset [1]. Prepubertal body composition has a critical effect on puberty onset because the conversion of androgen to estrogen occurs in adipose tissue, which is a significant source of extragonadal estrogen [8,9].

Girls who consumed more protein were likely to reach EMO. This may be relevant to the early protein hypothesis which demonstrates that early protein intake predisposes children to adiposity rebound at the time of preceding pubertal marker onset [13,24] that increases the leptin levels [1]. Animal protein intake was negatively associated with AAM (Figure 5). This weak association may be due to the wide range of 95% CI and/or the existence of heterogeneity (*I*^2^ > 50%), possibly caused by the different times of dietary assessment among the three included studies [13,17,24] (Table 1). Animal protein stimulates insulin-like growth factor 1 (IGF-1) secretion, which causes the expression of gonadotropin-releasing hormone (GnRH), required for pubertal onset [36]. High meat and red meat intake have been associated with EMO in girls [19,34]. Red meat is a major source of zinc, iron, and vitamin B_12_. Rogers et al. [19] found a significant relationship between zinc intake and EMO. Higher plasma ferritin status and greater iron storage in middle childhood were also related to earlier AAM [37]. Concordantly, we observed that a high iron intake was associated with EMO. Besides animal protein, vegetable protein has been noticed to be negatively associated with EMO risk [14,17]. Additionally, a delay of AAM was observed when there was a high intake of grains, nuts, beans, legumes, and fruits, which are the primary sources of vegetable protein [16,17].

Fat intake is a strong predictor of earlier AAM, independent of body fat percentage [27]. However, there was a lack of statistical evidence of overall WMD of fat intake in relation to EMO. The fact is that the typical western diet, high in saturated fatty acids (FAs), is largely related to the increased prevalence of obesity, a critical factor which may lead to early puberty [9], and a high-fat diet during puberty may accelerate breast development [38]. Menarche represents the final physical event in a series of steps known as puberty [39] and typically occurs approximately two years after the onset of breast development [40]. Therefore, it is proposed that a high-fat diet may be associated with EMO because it accelerates breast development, the earliest secondary sexual characteristic in girls.

PUFA intake during early childhood has been found to influence puberty onset [41]. The dose-response effect of PUFA intake during late childhood was positively associated with EMO in the present study. PUFAs are essential FAs involved in the growth and reproductive procress through direct effects on steroidogenic machinery and mammary gland development [41]. In vitro, PUFAs modulate adrenal steroidogenesis and act on steroidogenesis-related transcription factors that affect steroid acute regulator protein expression [42]. Eventually, adrenal androgen stimulates GnRH neurons that are required for puberty onset [1]. Maclure et al. [27] revealed that only n-3 FAs were related to EMO in girls, not n-6 FAs. In contrast, an effect of n-6 PUFAs on puberty onset was discovered [43]. The ratio of n-6 to n-3 FAs is crucial to health. The timing of dietary exposure and the n-6 to n-3 FA ratio is related to puberty onset [41]. In vivo, an increased ratio of n-6 to n-3 FAs (5:1) modulated the reproductive function in female zebrafish, independent of the total dietary lipid levels [44]. The optimal n-6 to n-3 FA ratio for healthy puberty was beyond our study but merited further investigation.

The effect of MUFA intake during childhood on EMO has been inconsistent. A recent in vitro and in vivo study revealed that oleic acid, a MUFA, and the primary FA in olive oil, could affect puberty onset by stimulating mammary gland development and increasing the serum IGF-1 levels [45]. In contrast, we observed that MUFA intake was likely linked to delayed menarche onset underlying dose-response effects after pooling adjusted risk ratios from three studies conducted in Canada [16,18,26] and one conducted in the United States [27]. In Canada, the main dietary source of MUFA is canola oil, which accounted for three quarters of all processed vegetable oils during the 1990s [46]. Therefore, the type of MUFA intake, which protected girls from EMO in the present study, may be primarily from canola oil. Thus, more studies are warranted to assess the role of specific types of MUFAs on EMO.

A high carbohydrate intake contributes to excess EI and causes substantial weight gain, as stated, eventually influencing puberty onset [47]. In girls with BMI ≥ 18.5 kg/m^2^, we observed a high pooled WMD of carbohydrate intake in the EMO group than in the NEMO group, derived from unadjusted analysis. However, the carbohydrate was found to delay menarche onset, as determined using adjusted models (Figure 6). This may be explained by that carbohydrate could conceivably influence estrogen metabolism by depressing the formation of catechol estrogen from estradiol [48]. Remarkably, the overall WMD of carbohydrate intake in girls with EMO was lower than that in girls with NEMO among girls with BMI < 18.5 kg/m^2^, as determined from the data of Maclude et al. [27] and Tehrani et al. [28]. Noteworthy, malnutrition was found to be linked to a delay of AAM [49] that may not be solely attributed to the amount of carbohydrate intake.

We found that the dose-response effect of fiber intake in childhood was likely associated with a delayed menarche onset. Fiber intake has been considered to modulate circulating estrogen levels, which affects puberty onset mediated by the hypothalamus–pituitary–gonad system [1,17]. A cross-sectional study of 46 countries and areas discovered a strong positive correlation between fiber intake and AAM [50]. Higher intakes of insoluble fiber, cellulose fiber, and grain fiber, which are major sources of dietary fiber, were observed in girls who had later menarche onset [17,18]. Expressed in doses, consumption of either 18.19–21.81 g/day of fiber (quartile 1) or more than 25.48 g/day of fiber (quartile 4) was significantly linked to delayed menarche onset. Though the consumption of 21.81–25.48 g/day of fiber (quartile 3) did not show a significant association with EMO, it seems to approach the borderline significance (*p* = 0.07). However, Cheng et al. [22] denied the association between fiber intake and AAM, which included much lower doses (7.9–28.3 microgram/day) than that in the included studies [17,18]. In fact, the doses of fiber in our meta-analysis are much higher than the recommended level calculated by age plus 5 g/day [51]. These contrasting findings are believed to be due to Bertrand’s rule, which states that either health benefits or adverse consequences are associated with nutrient intake at either optimal intake or beyond this threshold [52]. Furthermore, dietary fiber-rich foods often contain high amounts of isoflavones [23]. Isoflavones are known to have antiestrogen effects because they inhibit the actions of enzyme aromatase and of 17 β-hydroxysteroid dehydrogenase and directly interact with estrogen receptors to limit endogenous estrone and estradiol synthesis [1]. Girls with a high intake of isoflavones entered puberty later [22]. However, soy-based infant formula (containing isoflavones) was not found to be associated with early puberty onset [53]. The timing effect of isoflavones on pubertal development warrants future research.

We found that girls with EMO consumed higher intake of carotene in childhood, which requires a further mechanistic explanation. Carotene was hypothesized in one prospective study to have antiestrogenic effects through inhibition of estrogen signaling via 17 β-estradiol that may protect girls from early puberty onset [54], as previously stated [1]. However, a very high carotene intake reduced fertility in cows [55], indicating the opposing dose-dependent effect [56] of carotene on reproductive function. Although the present study observed the weak association of vitamin C and magnesium with EMO in limited studies, the precise mechanism needs to be elucidated in further study.

### Strengths and Limitations

This is the first systematic review and meta-analysis to explain the association between EMO and nutrient intake during childhood. First, only studies with a longitudinal design were selected to explore the natural effects of energy and dietary factors on menarche onset. This reduced the possibility of outcome misclassification and reverse causation bias. Second, we carefully explained the effects of nutrient intake on EMO in girls whose BMI was in the normal range (18.5 to 19.6 kg/m^2^) in the included studies due to the fact that malnutrition was associated with a delay in AAM [49]. Third, various statistical methods were employed to ensure the robustness of our findings.

Some limitations of this study are addressed here. The dietary assessment methods differed between studies that limited the comparison of results and admitted measurement errors. Though most studies have controlled similar potential factors, such as mother’s AAM, parent education, age, height, body weight, and total EI, we could not contact authors to extract the RR values that were adjusted by the same confounders to attempt higher homogeneity across studies. In addition, we could not control the effect of modifiers of pubertal development, including birth weight [18], physical activity [3], and socioeconomic status [12,26]. Third, with respect to EMO, the recommended nutrient doses based on quartiles of nutrient intake could not be established because of a lack of relevant data. Fourth, we did not investigate the association between dietary quality, food groups, and EMO. However, it is important to identify the nutrients involved in disease etiology in order to isolate the true causative agents, and nutrient-focused research, enhances the mechanistic understanding of food and diet effects on EMO [10]. Finally, our findings may not be generalizable to all ethnicities, especially those in Eastern countries, because most of the selected studies were conducted in Western countries.

## 5. Conclusions

Caloric over-consumption and the association with EMO risk may be contributed by excess protein and carbohydrate intake during childhood in healthy girls. Through a high intake of animal proteins-, iron-, and PUFA-rich foods, girls are more likely to be EMO. In contrast, a high intake of fiber- and MUFA-enriched products is linked to a delay in menarche onset. Forthcoming studies are welcome to investigate the role of the specific type of MUFAs, PUFAs, and the n-6:n-3 PUFA ratios on EMO that may promote healthy sexual maturity in girls.

## Figures and Tables

**Table 1:** Characteristics of studies included in the systematic review.

Authors, Year, Country	Subjects(Mean ± SD/Age Range) (Yrs)	Dietary Method	Predictors (Nutrient Intake)		Follow-Up (Yrs)	Outcomes: AAM (Mean ± SD/Median, Min, Max) (Yrs)	Sample Size Menarche: Non-Menarche	Confounders	Main Findings
Kissinger and Sanchez, 1987, United States [34]	Premenarcheal girls aged 9–15	Multiple 24-h recalls Child reported	EI, intakes of carbohydrate, protein, fat, vitamins Food groups		>1	AAM Mother reported	230:770	NA NA	(1) No mean difference data between EMO and NEMO groups (2) Upper quartile intake of carbohydrate, thiamine, and iron was linked to 7–8-month later menarche compared with the lowest quartile (*p* < 0.05)
Moisan et al., 1990a, Canada [16]	2299 Caucasian girls aged 11.1 ± 0.6	3-Day dietary record Child reported	EI, intakes of macronutrients, SFAs, MUFAs, PUFAs, cholesterol, calcium, phosphorus, iron, crude fiber, and vitamins		1.4	12.1 (10.1–14.7) Mother reported	911:1388	Adjusted for age and mother’s AAM Adjusted for age and mother’s AAM	(1) No differences in mean intake of energy and macronutrients between menarche and premenarche groups (*p* > 0.05). Higher intake of vitamin C was linked to earlier AAM (2) Girls with the highest quartile of MUFA intake were likely to have early menarche
Moisan et al., 1990b, Canada [26]	666 Caucasia-n girls aged 9.5–12.5 years	3-Day dietary record Child reported	EI, intakes of macronutrients, saturated FAs, MUFAs, PUFAs, cholesterol, calcium, phosphorus, iron, crude fiber, and vitamins		1	11.4 (10.1–13.3) Mother reported	333:333	Adjusted for age and EI Adjusted for age and EI	(1) EMO girls consumed more energy, carbohydrate, lipids, MUFAs, iron, niacin, and vitamin A than NEMO girls (*p* < 0.05) (2) EI was positively associated with EMO in girls (OR = 1.5, 95% CI = 0.9–2.3) Girls with the highest MUFA and vitamin A intakes experienced menarche later (OR = 0.6, 95% CI = 0.4–0.9; OR = 0.5, 95% CI = 0.3–0.8, respectively)
Maclure et al., 1991, United States [27]	194 girls, aged 11.0±0.7	Semiquantitative FFQ Parent and child reported	Intakes of carbohydrate, sucrose, fiber, protein, total fat, FAs, cholesterol, and vitamins		4	12.5 Mother reported	74:120	NA Adjusted for baseline EI, height, and QI	(1) Early menarche was associated with higher intake of PUFAs, vitamins A, C, and B12, carotene, and retinol (*p* < 0.05) (2) The highest intake of saturated FAs, n-3 FAs, was associated with later menarche than the reference level (RR = 0.7, 95% CI = 0.4–1.2; RR = 2.7, 95% CI = 1.6–4.6, respectively). The highest intake of vitamin A was linked to AAM compared with the reference (RR = 1.3, 95% CI = 0.7–2.3)
de Ridder et al., 1991, Netherla-nds [17]	63 girls aged 9.6±0.04	7-Day food record Child reported	EI, intakes of fiber, grain fiber, vegetable fiber, protein fat, carbohydrate		3	14.3 Child reported	31:29	NA Adjusted for EI, height, dietary methods, timing, poly-saccharides	(1) No mean difference data between EMO and NEMO groups (2) Vegetable protein and fiber intakes were inversely associated with earlier AAM (β = −3.3 (1.5); β = −2.6 (1.2), respectively)
Merzenich et al., 1993, Germany [20]	261 girls aged 8–15	Semiquantitative FFQ; Parent and child reported	Intakes of carbohydrate, protein, and fat		2	9.7–15.6 Parent and child reported	94:167	NA Adjusted by age and total EI	(1) No mean difference data between EMO and NEMO groups (2) The highest quartile accelerated menarche onset (RR = 2.1; 95% CI = 1.1–4.0)
Petridou et al., 1996, Greece [12]	345 girls, aged 9–16	FFQ Child reported	EI, intakes of carbohydrate, protein, saturated FAs, MUFAs, and PUFAs		1	13 (9.9–16.0) Mother reported	166:179	NA Adjusted for total EI	(1) No mean difference data between EMO and NEMO groups (2) Girls with higher PUFA intake reached menarche later than those with the reference intake (RR= 0.79, 95% CI = 0.65–0.95)
Koprowski et al. 1999, United States [21]	1378 girls aged 8–13	7-Day FFQ Child reported	Total EI and intakes of carbohydrate, protein, and fat		4	9.7–14.8 Parent reported	464:215	NA Adjusted for age at dietary assessment, height, QI, EI, and ethnicity	(1) No mean difference data between EMO and NEMO groups (2) Higher EI was inversely associated with AAM (*p* trend < 0.05). Carbohydrate, protein, and fat intakes were unrelated to AAM (*p* < 0.05)
Berkey et al., 2000, United States [13]	67 Caucasian girls aged 3–5 or 6–8	Dietary history interview regarding the preceding 6 months Mother reported		EI, intakes of animal protein, vegetable protein, and total fat	4.83 and 8.83	12.8 ± 1.1 Medical records	NA	NA Adjusted for age and EI	(1) No mean difference data between EMO and NEMO groups (2) Vegetable protein intake at age 3–5 years was linked to delayed AAM (β = 2.19 (0.91)). Animal protein intake at age 6–8 years was linked to earlier AAM (β = −1.69 (0.76))
Koo et al., 2002, Canada [18]	637 girls aged 9.7±1.2	Semiqu-antitative FFQ Mother reported	Intakes of fiber, fat, and FAs		3	13.6 (8.5–15.6) Mother reported	187:402	NA Adjusted for EI, age at entry, time-dependent age at entry, body weight, birth weight, the logarithm of EI, mother’s AAM, and father’s career	(1) No mean difference data between EMO and NEMO groups (2) The fiber intake was negatively associated with EMO (HR = 0.54, 95% CI = 0.31–0.94). Increased cellulose and animal fat intakes were negatively associated with menarche onset (*p* trend = 0.009 and 0.03, respectively).
Cheng et al., 2010, Germany [22]	119 girls, aged 7.2 ± 1.0	3-Day dietary record	Intakes of isoflavones and dietary fiber at 1 and 2 years before age at take-off		1–2	12.6 ± 1.0 to 13.1 ± 1.2 Parent and child reported	108:11	NA Adjusted for smoking, baseline isoflavone intake, and baseline EI z-score	(1) No mean difference data between EMO and NEMO groups (2) Fiber and isoflavone intakes were not associated with AAM in the unadjusted model or adjusted model (*p*_trend = 0.4)
Gunther et al., 2010, Germany [14]	112 children (57 girls, 50.9%)	3-Day weighed dietary record for girls aged 3–4 or 5–6 years Parent reported	Intakes of protein, animal protein, and vegetable protein		Average = 9.3 and 7.3	12.8 ± 1.2	47:10	NA Adjusted for sex, EI, breastfeeding, birth year, and father’s university degree	(1) No mean difference data between EMO and NEMO groups (2) Higher animal protein intake at 5–6 years was related to earlier menarche (*p* trend = 0.02). Children with higher vegetable protein intake experienced later menarche (*p* trend = 0.02–0.03)
Remer et al., 2010, Germany [24]	109 German children (55 girls)	3-Day weighed dietary record Child reported	Intake of animal protein		1 and 2 before ATO	13.1 ± 0.8 Parent and child reported	NA	NA Adjusted for sex, EI, fat mass index, gestational age, birth weight, breastfeeding, and maternal overweight	(1) No mean difference data between EMO and NEMO groups (2) Animal protein intake was negatively associated with AAM (*p* = 0.07)
Roger et al., 2010, England [19]	3298 girls aged 12.9±0.2	FFQ at ages 3 and 7 years. 3-Day weight dietary record at age 10–11 years, Child reported	EI, intakes of total fat, saturated FAs, MUFAs, PUFAs, starch, sugar, total protein, animal protein, vegetable protein, fiber, vitamins		3.5	12.8 ± 0.2 Clinic reported	951:1419	NA Adjusted for BMI and height at the time of diet measurement	(1) At 3 years, protein, animal protein, and carotene intakes were higher in girls with EMO (*p* < 0.05). At 7 years, PUFA, protein, animal protein, and Zn intakes were higher in girls with EMO (*p* = 0.019). At 10 years, EI was higher in girls with EMO (*p* = 0.002) (2) At aged 3 and 7 years, protein, animal protein PUFA intakes were positively associated with EMO, but not at 10 years of age
Tehrani et al., 2013, Iran [28]	134 prepubertal girls aged 8.9 ± 2.4	24-h Dietary recalls Child reported	EI, intakes of carbohydrate, fat, protein, magnesium, phosphorus, milk, yogurt, and cheese		Medi-an follow-up = 6.5	12.7 ± 1.3 Child reported	60:74	NA Adjusted for EI, protein intake, the interval between the age at study initiation and the AAM, and mother’s AAM	(1) No significant differences in EI or carbohydrate, protein, or total fat intake between EMO and NEMO groups (*p* < 0.05) (2) Girls with EMO had higher intakes of calcium (OR = 3.20, 95% CI = 1.39–7.42), magnesium (OR = 2.43, 95% CI = 1.12–5.27), and phosphorus (OR = 3.37, 95% CI = 1.44–7.87) than girls with NEMO.
Cheng et al., 2019, Australia [15]	142 prepuber-tal children aged 8	3-Day food record Parent and child reported	EI, intakes of carbohydrate, fat, total protein, and animal protein		5–8	12.6 ± 1.0 Child reported	92:50	NA Adjusted for total energy, birth weight, height, zBMI, and mother’s occupation	(1) No mean difference data between EMO and NEMO groups (2) Lower absolute (*p* = 0.04) and energy-adjusted (*p* = 0.03) protein intake was linked to EMO. Lower dietary protein (relative to carbohydrate and fat) intake consistently predicted EMO. Animal protein intake was not associated with menarche onset

*Definition of abbreviations*: AAM, age at menarche; BMI, body mass index; EMO, earlier menarche onset; EI, energy intake; FAs, fatty acids; FFQ, food frequency questionnaire; NA, not applicable; NEMO, non-early menarche onset; PUFA, polyunsaturated fatty acid; QI, Quetelet’s index; RR, relative risk; SD, standard deviation; zBMI, z-score BMI.

**Table 2:** Mean differences in nutrient intake between girls with EMO and NEMO.

Energy and Nutrient Intake	EMO (N)	NEMO (N)	Weighted mean Difference (WMD, 95% CI)		*I* ^2^
			Fixed Effect	Random Effect	
Energy (kcal/day)	2017	3230	60.2 (40.9, 79.6) *	51.7 (-1.1, 104.5)	68% *
Carbohydrate (g/day)	1398	2163	−0.1 (−3.7, 3.5)	−2.8 (−22.2, 16.7)	92%
Carbohydrate (% energy)	1398	2163	−0.1 (−3.8, 0.8)	−0.1 (−3.8, 0.8)	0%
Total fiber (g/day)	1172	1910	0.0 (−0.1, 0.1)	0.0 (−0.1, 0.1)	0%
Protein (g/day)	2349	3582	0.9 (0.3, 1.5) *	0.2 (−1.4, 1.8)	62% *
Protein (% energy)	2349	3582	0.2 (0.1, 0.3) *	0.04 (−0.3, 0.3)	56%
Fat (g/day)	1398	2163	1.2 (0.02, 2.4) *	1.8 (−0.7, 4.2)	34%
Fat (% energy)	1398	2163	-0.8 (-1.1, -0.5) *	-0.03 (−1.0, 0.9)	73% *
Saturated FAs (g/day)	1338	2089	0.2 (−0.4, 0.8)	0.3 (−1.4, 2.1)	60%
MUFAs (g/day)	1338	2089	0.5 (0.04, 1.0) *	0.5 (−0.3, 1.4)	18%
PUFAs (g/day)	2289	3508	0.4 (0.2, 0.6) *	0.5 (0.2, 0.8) *	21%
Cholesterol (g/day)	1172	1910	1.9 (−4.3, 8.1)	1.9 (4.3, 8.1)	0%
Calcium (mg/day)	1404	1649	−2.4 (−31.5, 26.7)	−2.4 (−31.5, 26.7)	0%
Phosphate (mg/day)	971	1462	8.9 (−17.5, 35.3)	8.9 (−17.5, 35.3)	0%
Magnesium (mg/day)	679	1141	3.1 (0.3, 5.9) *	3.1 (0.3, 5.9) *	0%
Iron (mg/day)	911	1388	0.2 (−0.1; 0.4)	NA	NA
Vitamin B1 (mg/day)	985	1508	0.0 (−0.03, 0.03)	0.0 (−0.03, 0.03)	0%*
Riboflavine (µg/day)	985	1508	−1.0 (−1.0, −0.9) *	−0.4 (−1.6, 0.7)	96% *
Vitamin B3 (µg/day)	985	1508	0.2 (−0.3, 0.6)	0.2 (−0.3, 0.6)	0%
Vitamin B6 (mg/day)	985	1508	0.0 (−0.03, 0.03)	0.0 (−0.03, 0.03)	0%
Vitamin B9 (µg/day)	985	1508	−0.7 (−7.8, 6.4)	−0.7 (−7.8, 6.4)	0%
Vitamin B12 (µg/day)	985	1508	0.01 (−0.1, 0.1)	0.2 (−0.4, 0.8)	59%
Vitamin C (mg/day)	1418	1695	5.4 (1.1, 9.7) *	8.6 (−9.7, 26.9)	23%
Vitamin A (IU/day)	1418	1695	−83.8 (−218.3,50.7)	1476.9 (−2262.5, 5261.4)	81% *
Carotene (µg/day)	1068	1600	78.1(13.9, 142.4) *	744.7 (−992.9, 2482.2)	72%
Vitamin D (IU/day)	985	1508	−0.5 (−10.4, 9.4)	−0.5 (−10.4, 9.4)	0%
Vitamin E (mg/day)	985	1508	0.1 (−0.1, 0.3)	0.1 (−0.1, 0.3)	0%

* *p* < 0.05. *Definition of abbreviations*: CI, confidence interval; EMO, earlier menarche onset; FAs, fatty acids; MUFAs, monounsaturated fatty acids; NA, not applicable; NEMO, non-early menarche onset; PUFAs, polyunsaturated fatty acids; WMD, weighted mean difference.

## Supplementary Materials

The following are available online at https://www.mdpi.com/2072-6643/12/9/2544/s1, Table S1: PECOS criteria for inclusion and exclusion criteria, Table S2: Quality assessment of the included studies using a modified Quality Assessment Tool for Systematic Reviews of Observational Studies (QATSO) Score for assessing relationship between prepubertal nutrient intake and EMO, Table S3: Analyses of publication bias using Egger Test for assessing relationship between prepubertal nutrient intake and EMO, Table S4: Univariate meta-regression analyses, Table S5: Association between EMO and quartile of nutrient intake in childhood, Figure S1: Forest plot of mean differences of carbohydrate, total fat, and protein intake between EMO and NEMO among girls with BMI < 18.5 kg/m^2^, Figure S2: Association between EMO and quartile of nutrient intake in childhood.
