# Supplementary material for: Nutrient Intake through Childhood and Early Menarche Onset in Girls: Systematic Review and Meta-Analysis
Source: Nutrients. 2020 Aug 22;12(9):2544. doi: 10.3390/nu12092544 (PMC7551779; doi:10.3390/nu12092544)
Supplement: Supplementary file 1 [file nutrients-12-02544-s001.pdf]

Supplemental Materials

Table S1 PECOS criteria for inclusion and exclusion criteria 2
Table S2 Quality assessment of the included studies using a modified Quality Assessment Tool for Systematic Reviews of Observational Studies (QATSO) Score for assessing relationship between prepubertal nutrient intake and EMO 5
Table S3 Analyses of publication bias using Egger Test for assessing relationship between prepubertal nutrient intake and EMO 11
Table S4 Univariate meta-regression analyses 12
Table S5 Association between EMO and quartile of nutrient intake in childhood 13
Figure S1 Forest plot of mean differences of carbohydrate, total fat, and protein intake between EMO and NEMO among girls with BMI < 18.5 kg/m^2. 15
Figure S2 Forest plot of mean differences of fiber intake between EMO and NEMO among girls 16

Table S1 PECOS criteria for inclusion and exclusion criteria

Criteria	Inclusion criteria	Exclusion criteria
Participants	Healthy girls who had not yet reached menarche onset at the age of dietary assessment.	Girls with diseases that affected pubertal development (e.g., congenital gonadal dysplasia or iodine deficiency disorder)
Exposure	Dietary nutrients, such as EI and intakes of carbohydrate, protein, fat, and vitamins.	Dietary pattern, food items
Comparison	Nutrient intake between EMO and NEMO	No statistical association between nutrient intake and EMO
Outcome	Menarche onset, AAM	Other pubertal markers (growth spurt, thelarche, or pubic hair)
Study design	Cohort studies, longitudinal studies, prospective studies	Cross-sectional studies, interventional studies, systematic reviews, meta-analyses

Definition of abbreviations: AAM, age at menarche; EI, energy intake; EMO, earlier menarche onset; NEMO, non–earlier menarche onset.

Table S2 Quality assessment of the included studies using a modified Quality Assessment Tool for Systematic Reviews of Observational Studies (QATSO) Score for assessing relationship between prepubertal nutrient intake and EMO

Author, year	External validity	Reporting			Bias	Confounders	Total (Maximum 6 points)	Quality rating of study
	Representati-veness of sampling method	Clearly defined eligibility criteria	Pre-menarcheal Dietary assessment	Standardized Dietary assessment	Clearly defined outcome examine	Statistical tests		
Kissinger&Sanchez, 1987	*	-	*	*	-	-	3	Satisfactory
Moisan et al., 1990	*	-	*	*	*	*	5	Good
Moisan et al., 1990b	*	-	*	*	*	*	5	Good
Maclure et al., 1991	-	-	*	*	-	*	3	Satisfactory
Ridder et al., 1991	-	*	*	*	*	*	5	Good
Merzenich et al., 1993	*	-	*	-	-	*	3	Satisfactory
Petridou et al., 1996	*	*	*	-	*	*	5	Good
Koprowski et al., 1999	*	*	*	-	-	*	4	Satisfactory
Berkey et al, 2000	-	-	*	*	-	*	3	Satisfactory
Koo et al., 2002	*	*	*	*	*	-	5	Good
Cheng et al, 2010	-	*	*	-	*	*	5	Good
Gunther et al, 2010	-	*	*	*	-	*	4	Satisfactory
Remer et al., 2010	-	*	*	-	-	*	3	Satisfactory
Rogers et al., 2010	*	-	*	*	*	*	5	Good
Tehrani et al., 2013	*	*	*	*	-	*	5	Good
Cheng at al., 2019	-	-	*	-	*	*	3	Satisfactory

** represents for answer with "yes" or 1 point.*

Definition of abbreviation: EMO, early menarche onset.

Table S3 Analyses of publication bias using Egger Test for assessing relationship between prepubertal nutrient intake and EMO

Macronutrients	Est.	±SE	Slope	t test	df	*P-value*
Energy (kcal/day)	−0.7	± 1.2	72.8	−0.7	4	0.6
Carbohydrates (g/day)	−1.1	± 2.3	3.5	−0.4	3	0.7
Protein (g/day)	−0.8	± 0.9	1.3	−0.8	4	0.4
Fat (g/day)	−2.7	± 1.9	6.9	−1.4	4	0.2
Saturated FAs (g/day)	0.1	± 1.5	0.1	−0.1	2	0.9
MUFAs (g/day)	−0.1	± 1.0	0.6	−0.1	2	0.9
PUFAs (g/day)	0.9	± 0.7	0.2	1.3	3	0.3

Definition of abbreviation: df = degrees of freedom; Est., estimate; SE, standard; EMO, early menarche onset; FAs, fatty acids; MUFAs, monounsaturated fatty acids; NA, not applicable; PUFAs, polyunsaturated fatty acids.

Table S4 Univariate meta-regression analyses

Nutrient intakes	Country		Age#(yrs)		BW#(kg)		BMI #(kg/m^2)		Maternal AAM (yrs)		Dietary methods (DR vs FFQ)	
	OR	p-value	β	p-value	β	p-value	β	p-value	β	p-value	OR	p-value
Energy (Kcal/day)	NS		15.9	0.6	21.4	0.4	61.2	0.05*	-27.0	0.6	-18.4	0.8
Carbohydrates (g/day)	NS		-12.2	0.4	NA		10.5	0.01*	-25.3	0.6	-21.2	0.3
Fiber (g/day)	NS		-0.7	0.1	NA		NA		NA		-0.9	<0.01*
Protein (g/day)	NS		-0.03	0.9	0.1	0.9	1.6	<0.01*	-1.1	0.6	-1.9	0.5
Fat (g/day)	NS		-1.2	0.6	NA		0.3	0.9	-3.1	0.3	-0.5	0.9
Saturated FA (g/day)	NS		-1.8	0.2	NA		0.9	0.7	NA		-0.2	0.9
MUFAs (g/day)	NS		-0.7	0.5	NA		0.9	0.5	NA		0.03	0.9
PUFAs (g/day).	NS		-0.1	0.6	0.2	0.2	0.3	0.4	0.3	0.3	-0.6	0.1
Cholesterol (g/day)	NS		-3.1	0.7	NA		NA		NA		1.3	0.9

#evaluated at the time of dietary assessment.

*$P < 0.05$.

Definition of abbreviation: AAM, age at menarche; BMI, body mass index; β, beta regression; BW, body weight; DR, dietary record; EMO, early menarche onset; FA, fatty acid; FFQ, food frequency questionnaire; MUFAs, monosaturated fatty acids; NA, not applicable; NEMO, non-early menarche onset; NS, non-significant; OR, odds ration; PUFA, polyunsaturated fatty acid; yrs, years.

Electronic Supplemental Materials

Table S5 Association between EMO and quartile of nutrient intake in childhood

Nutrient intakes	No. studies	Quartile 1	Quartile 2 RR (95%CI)	Quartile 3 RR (95%CI)	Quartile 4 RR (95%CI)
Energy intake (kcal/day)					
Fix-effect model	5	1 (ref)	0.95 (0.83, 1.08)	0.99 (0.86, 1.13)	1.02 (0.88, 1.18)
Random effect model	5	1 (ref)	0.97 (0.82, 1.15)	0.96 (0.78, 1.20)	1.04 (0.80, 1.36)
			I^2=30%, p=0.22	I^2=50.5%, p=0.08	I^2=63.7%, p=0.03
Carbohydrate (g/day)					
Fix-effect model	3	1 (ref)	0.81 (0.71, 0.94)*	0.98 (0.87, 1.11)	0.98 (0.85, 1.13)
Random effect model	3	1 (ref)	0.81 (0.71, 0.94)*	0.96 (0.79, 1.17)	0.98 (0.85, 1.13)
			I^2=0%, p=0.59	I^2=44.4%, p=0.17	I^2=0%, p=0.68
Fiber (g/day)					
Fix-effect model	3	1 (ref)	0.86 (0.75, 1.00)*	0.88 (0.76, 1.01)	0.83 (0.69, 1.00)*
Random effect model	3	1 (ref)	0.83 (0.67, 1.03)	0.88 (0.76, 1.01)	0.80 (0.61, 1.04)
			I^2=27.4%, p=0.25	I^2=0%, p=0.83	I^2=30.6%, p=0.24
Protein (g/day)					
Fix-effect model	3	1 (ref)	1.01 (0.87, 1.18)	1.14 (1.00, 1.31)*	1.07 (0.90, 1.26)
Random effect model	3	1 (ref)	1.01 (0.87, 1.18)	1.14 (1.00, 1.31)*	1.07 (0.90, 1.26)
			I^2=0%, p=0.72	I^2=0%, p=0.83	I^2=0%, p=0.68
Total Fat (g/day)					
Fix-effect model	4	1 (ref)	1.06 (0.94, 1.18)	1.07 (0.92, 1.24)	1.07 (0.92, 1.23)
Random effect model	4	1 (ref)	1.06 (0.85, 1.33)	1.07 (0.92, 1.24)	1.07 (0.92, 1.23)
			I^2=60.1%, p=0.05	I^2=0%, p=0.40	I^2=0%, p=0.41
Saturated FAs (g/day)					
Fix-effect model	4	1 (ref)	1.03 (0.9, 1.18)	1.02 (0.88, 1.18)	0.99 (0.85, 1.16)
Random effect model	4	1 (ref)	0.85 (0.6, 1.21)	1.00 (0.85, 1.18)	0.90 (0.70, 1.16)
			I^2=75.4%, p<0.01	I^2=7.3%, p=0.35	I^2=39.5%, p=0.18
MUFAs (g/day)					
Fix-effect model	4	1 (ref)	0.87 (0.72, 1.06)	0.74 (0.60, 0.92)*	0.66 (0.50, 0.86)*
Random effect model	4	1 (ref)	0.86 (0.66, 1.11)	0.74 (0.59, 0.94)*	0.66 (0.50, 0.86)*
			I^2=39.6%, p=0.17	I^2=13.1%, p=0.32	I^2=0%, p=0.79
PUFAs (g/day)					
Fix-effect model	4	1 (ref)	1.14 (0.98, 1.32)	1.10 (0.95, 1.28)	1.25 (1.05, 1.49)*
Random effect model	4	1 (ref)	1.14 (0.98, 1.32)	1.10 (0.95, 1.28)	1.37 (1.03, 1.81)*
			I^2=0%, p=0.78	I^2=0%, p=0.96	I^2=44.0%, p=0.15
Calcium (mg/day)					
Fix-effect model	2	1 (ref)	1.08 (0.91, 1.28)	0.90 (0.78, 1.04)	1.00 (0.83, 1.20)
Random effect model	2	1 (ref)	1.08 (0.91, 1.28)	0.90 (0.78, 1.04)	1.00 (0.83, 1.20)
			I^2=0%, p=0.46	I^2=0%, p=1.0	I^2=0%, p=1.0
Phosphate (mg/day)					
Fix-effect model	2	1 (ref)	0.89 (0.76, 1.05)	0.96 (0.80, 1.15)	1.01 (0.86, 1.20)
Random effect model	2	1 (ref)	0.79 (0.48, 1.30)	0.96 (0.80, 1.15)	0.91 (0.59, 1.41)
			I^2=85%, p<0.01	I^2=0%, p=0.36	I^2=76.4%, p=0.04
Iron (mg/day)					
Fix-effect model	2	1 (ref)	1.12 (0.92 1.36)	1.11 (0.96, 1.30)	1.20 (1.03, 1.40)*
Random effect model	2	1 (ref)	1.12 (0.92 1.36)	1.11 (0.96, 1.30)	1.20 (1.03, 1.40)*
			I^2=0%, p=0.72	I^2=0%, p=0.69	I^2=0%, p=1.0

Electronic Supplemental Materials

Vitamin B1 (mg/day)					
Fix-effect model	2	1 (ref)	0.92 (0.78, 1.08)	1.17 (1.0, 1.37)*	1.00 (0.84, 1.20)
Random effect model	2	1 (ref)	0.92 (0.78, 1.08)	1.17 (1.0, 1.37)*	1.00 (0.84, 1.20)
			I^2=0%, p=0.64	I^2=0%, p=0.46	I^2=0%, p=1.0
Riboflavine (mg/day)					
Fix-effect model	2	1 (ref)	1.03 (0.86, 1.24)	1.03 (0.86, 1.24)	1.00 (0.83, 1.20)
Random effect model	2	1 (ref)	1.03 (0.86, 1.24)	1.03 (0.86, 1.24)	1.00 (0.83, 1.20)
			I^2=0%, p=0.45	I^2=0%, p=0.45	I^2=0%, p=1.0
Vitamin B3 (mg/day)					
Fix-effect model	2	1 (ref)	1.01 (0.83, 1.23)	1.03 (0.86, 1.24)	1.00 (0.83, 1.20)
Random effect model	2	1 (ref)	0.92 (0.60, 1.42)	1.03 (0.86, 1.24)	1.00 (0.83, 1.20)
			I^2=67.7%, p=0.07	I^2=0%, p=0.44	I^2=0%, p=1.0
Vitamin B9 (mg/day)					
Fix-effect model	2	1 (ref)	0.90 (0.78, 1.04)	1.03 (0.86, 1.24)	1.00 (0.83, 1.20)
Random effect model	2	1 (ref)	0.90 (0.78, 1.04)	1.03 (0.86, 1.24)	1.00 (0.83, 1.20)
			I^2=0%, p=1.0	I^2=0%, p=0.45	I^2=0%, p=1.0
Vitamin B12 (µg/day)					
Fix-effect model	3	1 (ref)	1.03 (0.88, 1.21)	1.03 (0.88, 1.22)	1.00 (0.83, 1.19)
Random effect model	3	1 (ref)	1.03 (0.88, 1.21)	1.03 (0.88, 1.22)	1.00 (0.83, 1.19)
			I^2=0%, p=0.36	I^2=0%, p=0.89	I^2=26.9%, p=0.25
Vitamin C (mg/day)					
Fix-effect model	3	1 (ref)	1.00 (0.88, 1.14)	1.07 (0.89, 1.29)	1.10 (0.92, 1.32)
Random effect model	3	1 (ref)	1.00 (0.88, 1.14)	1.07 (0.89, 1.29)	1.10 (0.92, 1.32
			I^2=0%, p=0.44	I^2=0%, p=0.89	I^2=0%, p=1.0
Vitamin A (IU/day)					
Fix-effect model	3	1 (ref)	0.99 (0.86, 1.13)	1.04 (0.88, 1.22)	1.19 (1.01, 1.41)*
Random effect model	3	1 (ref)	0.99 (0.86, 1.13)	0.99 (0.78, 1.26)	1.74 (0.88, 3.47)
			I^2=0%, p=0.89	I^2=28.4%, p=0.25	I^2=87.4%, p<0.01
Vitamin D (IU/day)					
Fix-effect model	2	1 (ref)	1.14 (0.98, 1.33)	0.95 (0.77, 1.17)	0.97 (0.80, 1.16)
Random effect model	2	1 (ref)	1.03 (0.70, 1.51)	0.95 (0.77, 1.17)	0.97 (0.80, 1.16)
			I^2=65.9%, p=0.08	I^2=0%, p=0.37	I^2=0%, p=0.39

Definition of abbreviation: EMO, early menarche onset; FAs, fatty acids; MUFAs, monounsaturated fatty acids; PUFAs, polyunsaturated fatty acids.

Electronic Supplemental Materials

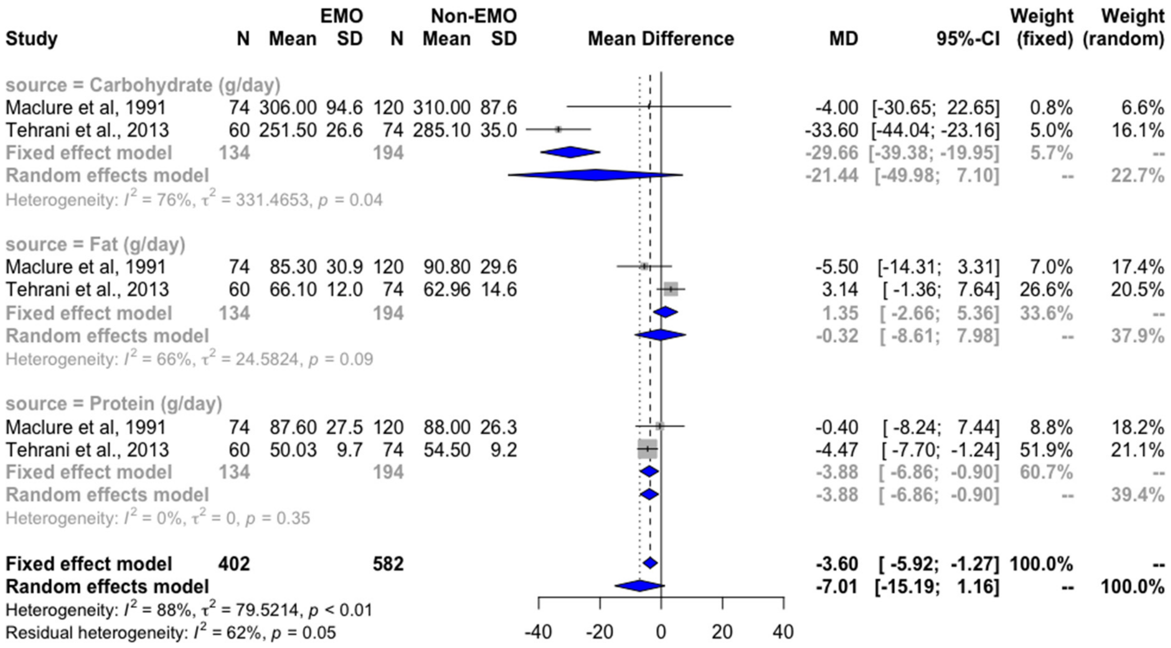

Figure S1 Forest plot of mean differences of carbohydrate, total fat, and protein intake between EMO and NEMO among girls with BMI < 18.5 kg/m^2.

Definition of abbreviation: EMO, early menarche onset; NEMO, non-early menarche onset; MD: mean difference.

Electronic Supplemental Materials

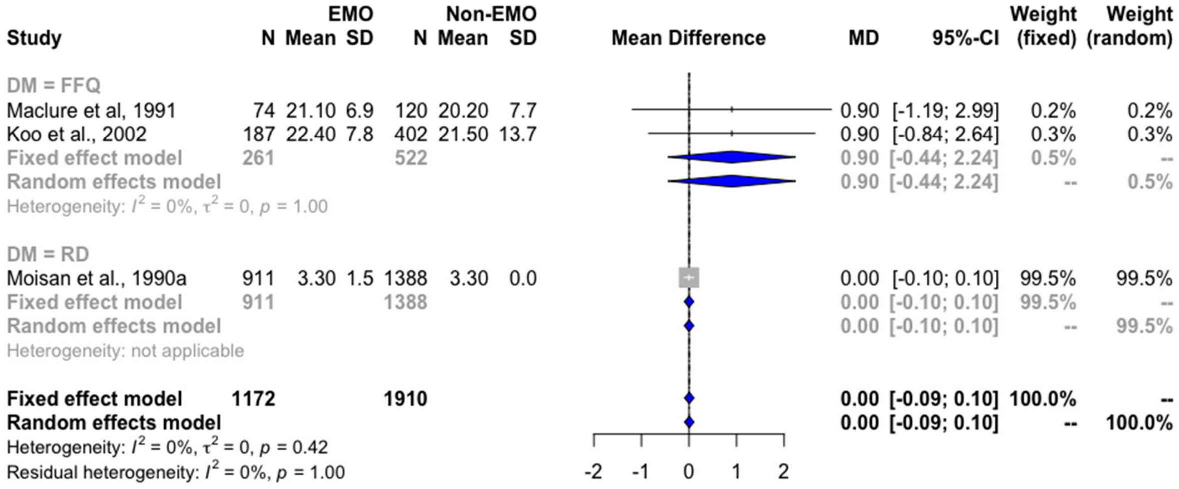

Figure S2 Forest plot of mean differences of fiber intake between EMO and NEMO among girls

Definition of abbreviation: EMO, early menarche onset; FFQ, food frequency questionnaire; DR, dietary record; NEMO, non-early menarche onset; MD: mean difference.
